# Chemical PARP Inhibition Enhances Growth of Arabidopsis and Reduces Anthocyanin Accumulation and the Activation of Stress Protective Mechanisms

**DOI:** 10.1371/journal.pone.0037287

**Published:** 2012-05-25

**Authors:** Philipp Schulz, Jenny Neukermans, Katrien Van Der Kelen, Per Mühlenbock, Frank Van Breusegem, Graham Noctor, Markus Teige, Michael Metzlaff, Matthew A. Hannah

**Affiliations:** 1 Bayer CropScience NV, Gent, Belgium; 2 Department of Biochemistry and Cell Biology, MFPL, University of Vienna, Vienna, Austria; 3 Institut de Biologie des Plantes, Université de Paris Sud XI, Orsay, France; 4 VIB Department of Plant Systems Biology, Ghent University, Ghent, Belgium; 5 Department of Plant Biotechnology and Bioinformatics, Ghent University, Ghent, Belgium; Lawrence Berkeley National Laboratory, United States of America

## Abstract

Poly-ADP-ribose polymerase (PARP) post-translationally modifies proteins through the addition of ADP-ribose polymers, yet its role in modulating plant development and stress responses is only poorly understood. The experiments presented here address some of the gaps in our understanding of its role in stress tolerance and thereby provide new insights into tolerance mechanisms and growth. Using a combination of chemical and genetic approaches, this study characterized phenotypes associated with PARP inhibition at the physiological level. Molecular analyses including gene expression analysis, measurement of primary metabolites and redox metabolites were used to understand the underlying processes. The analysis revealed that PARP inhibition represses anthocyanin and ascorbate accumulation under stress conditions. The reduction in defense is correlated with enhanced biomass production. Even in unstressed conditions protective genes and molecules are repressed by PARP inhibition. The reduced anthocyanin production was shown to be based on the repression of transcription of key regulatory and biosynthesis genes. PARP is a key factor for understanding growth and stress responses of plants. PARP inhibition allows plants to reduce protection such as anthocyanin, ascorbate or Non-Photochemical-Quenching whilst maintaining high energy levels likely enabling the observed enhancement of biomass production under stress, opening interesting perspectives for increasing crop productivity.

## Introduction

Plants have to survive their surrounding environment by counteracting the constant challenges posed by biotic and abiotic stress. They are able to do this by activating signaling pathways which modulate downstream response pathways in order to protect themselves in the short-term and adapt in the longer-term. These pathways are targets for developing approaches to increase stress resistance of crops and reduce yield losses. In the last decade, intensive studies have revealed a complex network of signaling pathways involved in abiotic stress responses. Widespread changes of transcripts and metabolites were observed in many kinds of stress, including for example osmotic [1] and heat stress [2]. These studies have shown that in *Arabidopsis* a number of response pathways, such as reactive oxygen species (ROS) and abscisic acid (ABA) signaling, are conserved between many abiotic stresses [for review see 3], [4], [5], whilst other components are more specific. Among the conserved responses transcription factors such as ZAT12 or detoxification enzymes like ascorbate peroxidase 2 (APX2) which are induced by multiple abiotic stresses are thought to play a central role [6], [7]. The potential of transcription factors to increase stress resistance and yield has attracted interest for translating findings from *Arabidopsis* to crops, for example CBF3 in rice [8] or NF-YB in maize [9].

A typical downstream response to abiotic stress conditions, such as high light or cold, is the induction of genes that encode enzymes involved in the production of secondary metabolites, for instance, flavonols [10], [11]. Among these metabolites anthocyanins form one of the most prominent groups and are often involved in stress responses [12], [13]. Anthocyanins, which are only present in plants, could act as ROS protective agents [14], [15], [16]. However, the anthocyanin synthesis pathway genes are not only induced by ROS, but also by several other signals like the deprivation of nutrients, including phosphorus and nitrogen, or high concentrations of exogenous sugars, particularly sucrose [17], [18], [19]. How these signals are transmitted and which signaling pathways are involved is currently not fully understood. A part of the signals can be assigned to the CRYPTOCHROME1 (CRY1)-dependent light signaling pathway [20]. Additionally, a hormonal pathway including ABA as well as jasmonate and gibberellins was shown to contribute to the regulation of anthocyanin biosynthesis [21]. These signaling pathways are controlled by MYB and bHLH transcription factors, which can control the main steps of the pathway, as shown for *Production Of Anthocyanin Pigment 1* (*PAP1/Myb75*), or more specific steps, like *Transparent Testa 8* (*TT8*) which controls the expression of *Dihydro-flavonol reductase* (*DFR*) [22], [23], [24].

As described above different mechanisms are involved in the defense against abiotic stresses. In this context one group of enzymes demonstrated to have a key function in the protection against negative effects of environmental stress is the poly(ADP-ribose)-polymerases (PARPs). PARP was first described in plants 15 years ago [25], [26], [27] and is found exclusively in eukaryotes. These proteins are characterized by the so called PARP signature motive [28], [29] and modify their target proteins post-translationally by adding ADP-ribose polymers (PAR) to lysine residues. The ADP-ribose is derived from nicotinamide adenine dinucleotide (NAD+) [30], leading to its conversion to nicotinamide. This destructive use of NAD+ as a substrate links PARP activity with cellular energy homeostasis and consequently with cell death processes [27]. In general the function of PARPs and poly(ADP-ribose) (PAR) is less well described in plants compared to animals [31], [32]. In mammals, the 18 PARPs so far described [33] are linked with additional processes like DNA damage repair, transcriptional regulation and chromatin modifications [for review 34], [35]. So far nine proteins with a PARP signature were identified in *Arabidopsis* but six of them, such as Radical Induced Cell Death 1 (RCD1), do not have a catalytic domain and no PARP activity was found [36], [37]. Of the three Arabidopsis PARPs that have catalytic activity (PARP1-3), PARP1 and PARP2 are mainly assigned to tolerance of abiotic [38], [39] and biotic stress [40], but they also have been implicated in developmental processes [41]. PARP1 and PARP2 are associated with DNA repair [38], [39] and transcriptional regulation [42], [43], [44]. PARP3, a more recently identified PARP, is induced by several abiotic stress and developmental cues, for example during seed development [41]. PARP also affects the level of ABA and consequently ABA related signaling [44]. Down-regulation of PARP activity increases resistance against abiotic stresses such as temperature, excessive light and drought [27], [39], [44], [45] and this resistance is correlated with a reduced poly(ADP-ribose) level [39]. A similar situation was observed earlier in animal cells [46].

How reduced PARP activity leads to the described effects still remains poorly understood. One possibility is through altered NAD+ metabolism [47], whereby lower NAD+ consumption leads to a lower need for the highly energy dependent recycling pathway [39]. This allows the NAD+ and ATP pools to be maintained and avoids the link to cell death pathways where NAD+ and ATP depletion are major inductive signals [48], [49]. Another possibility is that PARP regulates key stress signaling pathways at the transcriptional level [44], by direct control [42] or indirectly by changes in ABA related signaling [44]. A third potential mode of action was recently described for cell cultures, showing that PARP activity and changes in the Redox status of cells are correlated within the cell cycle [50].

In this study we investigate the relation between PARP activity and anthocyanin accumulation which has not been studied so far despite the recognized importance of both in stress tolerance. Many abiotic stress conditions, such as cold, excessive light or nutrient deprivation induce the production and accumulation of stress protective molecules, like anthocyanin pigments. The enzymes of the anthocyanin biosynthesis pathway are tightly regulated at the transcriptional level. This regulation is conferred by major transcription factors including Myb75/PAP1 and Myb90/PAP2, which control important bottlenecks such as Phenyalanin-ammonium-lyase (PAL), Chalcone synthase (CHS) or Dehydroflavonol reductase (DFR) and by other transcription factors, like Transparent Testa 8 (TT8), which have more specific functions. Here we investigate, the effects of chemical inhibition of PARP activity in *Arabidopsis* on stress tolerance and anthocyanin accumulation, both in response to long-term chloroplastic reactive oxygen stress and to high levels of exogenous sucrose. Detailed analysis of growth, photosynthesis, cellular redox status and the expression of anthocyanin biosynthetic genes show that chemical down-regulation of PARP activity reduces the accumulation of anthocyanin as well as ascorbate, alters photosynthesis and improves stress tolerance in whole plants. The reduction of anthocyanin accumulation is based on transcriptional regulation.

## Methods

### Growth conditions

T-DNA insertion lines for *PARP1*, *PARP2* and *PARP3* (Salk_097261C, Salk_145153C and Salk_108092C) and 35S:*PAP1* (Production of Anthocyanin Pigment 1) (At1g56650) over-expression line (kindly provided by S. Vanderauwera (VIB)), were grown in sterile in-vitro conditions on ½ Murashige-Skoog [51] with 1.2% glucose and in 80–100 µE light, at 22–23°C and a long day regime (16 h/8 h). For expression analysis, plants were grown on media according to [44], but with 80–100 µE of light. PARP inhibitors were added to the growth media in final concentration of 0.2 mM. Paraquat, salt and sucrose were added to the growth media to final concentrations of 0.1 µM, 75 mM and 150 mM, respectively. All chemicals were obtained from Sigma-Aldrich™. Short-term treatments where performed by growing plants on standard media in control conditions and transferring them to the respective treatments as indicated in the text.

### Growth analysis

Photographs of non-destructed plants were taken at the indicated time points and used to measure the total leaf area and rosette diameter with the ImageJ software. This data was subsequently used to calculate the average leaf area and rosette diameter of the plants.

### Expression analysis

For expression analysis RNA was extracted with the Plant RNAeasy Kit (Qiagen) following the manufacturer's instructions with additional on column DNase (Roche) treatment. cDNA synthesis was performed with 1 µg RNA and SuperScript II Reverse Transcriptase reagents (Invitrogen) according to the manufacturer's instructions. Primers were designed using PrimerExpress and the specificity confirmed via melting curve analysis. qRT-PCR was performed with the ABI-Fast 7200 Detection system (Applied Bioscience) in 96 well plates with SYBR-Green (Roche), following the manufacturer's instructions. The expression was calculated using Ct values, with *PP2A* as a reference gene as described [52].

### Chlorophyll a fluorescence measurements

The operating efficiency of PSII [Y_II = (Fm′−F)/Fm′], the proportion of open PSII reaction centers [qP = (Fm′−F)/(Fm′-Fo′)] and the non-photochemical quenching [NPQ = (Fm-Fm′)/Fm′] were measured after illumination of dark adapted plants, in an induction curve using chlorophyll a fluorescence in a MAXI IMAGING-PAM chlorophyll Fluorometer (Heinz Walz, Germany) in four independent experiments.

### Biochemical analyses

For the chlorophyll determination ∼25 mg leaf material was used for extraction in 1 ml chilled Ethanol, for 24 hours in the dark at 4°C. Photometric determination of the absorbance at 663 nm and 645 nm was used to determine chlorophyll a and chlorophyll b content according to [53]. Anthocyanin determination was done with ∼25 mg leaf material for extraction in 1 ml acidified Methanol (99∶1 (v/v) Methanol: HCl) for 24 hours in darkness at 4°C. Photometric determination of the absorbance at 530 nm and 657 nm and calculation of the relative anthocyanin content was done as described in [22]. These measurements were used for subsequent calculation of the average anthocyanin and chlorophyll content of the plants. Oxidized and reduced forms of ascorbate, glutathione and NAD(P)+ were measured using the plate-reader assay exactly as described in [54] and subsequently used to calculate the total amounts of ascorbate, glutathione and NAD(P)+. The low reduction states of the redox couples in the *in-vitro* grown plants relative to that observed for soil grown plants was confirmed in a additional independent blind experiment (data not shown) and has since been observed for other plants growing *in-vitro* (Noctor et. al., unpublished data). Using GC-MS, non-targeted metabolite profiling was performed according [55]. All sample material for redox and non-targeted metabolite profiling was harvested and immediately frozen in liquid nitrogen and stored until use at −80°C.

### Statistics

Statistical analyses were performed using linear-mixed models using the gls() and lme() functions implemented in the *nlme* R package (http://www.r-project.org). Where applicable, experiment, block and plate effects were included as random effects and contrasts of interest were made based on treatment, stress and genotype.

### Microarray

For expression analysis, plants were grown in control conditions, either without (−3 MB) or with (+3 MB) 3-Methoxybenzamide in two independent experiments. Within each experiment, leaf material was harvested from ∼10 seedlings from each of four replicate plates and pooled prior to RNA extraction. Hybridization and raw data collection were done at the VIB-Microarray Facility (MAF). Data were analyzed with the bioconductor software [56] for the R statistical environment (www.r-project.org). Quality control and RMA expression estimates were obtained using the *affy* package [57] whilst the coefficients of differential expression due to 3 MB treatment were obtained using the *limma* package [58]. The vector of coefficients was loaded into the MAPMAN software for visualization and the Wilcoxon rank sum test implemented in the software was used to identify pathways of interest [59]. Data are available in ArrayExpress under accession number [E-MTAB-896].

## Results

### Chemical PARP inhibition improves plant growth and abiotic stress tolerance

**Figure 1:**
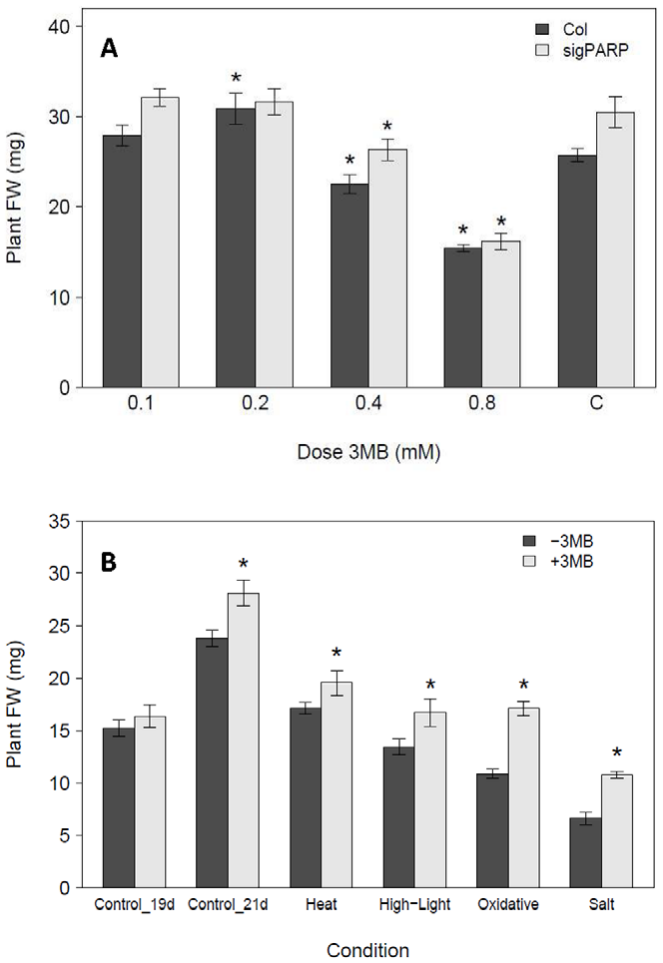
Dose response of plants to chemical PARP inhibition. **A**, *Arabidopsis thaliana* (Col-0) and PARPsig::RNAi (sigPARP) seedlings were grown for 21 days at 80–100 µE, 22°C on MS media containing different concentrations (mM) of the PARP inhibitor 3-Methoxy-benzamide (3 MB). The average individual fresh weight was determined by weighing 32 pooled seedlings from each plate, with four replicates (plates) in each experiment, repeated in two independent experiments (n = 8). Asterisks indicate significant (P<0.05) difference between Col-0 and PARPsig::RNAi seedlings grown without and those treated with PARP inhibitor. B, Arabidopsis plants were grown at 80–100 µE, 22°C on half MS medium and subjected to short- or long-term stress in the presence (+3 MB) or absence (−3 MB) of 0.2 mM of the PARP inhibitor 3 MB in the medium. For the short-term stress, 14 day old plants were transferred to either 450 µE (high-light) for two days and harvested at day 19 after three days of recovery or to 40°C (heat) for 6 h and harvested after seven days of recovery (day 21). For the long-term stress, plants were grown for 21 days either in control, 0.1 µM Paraquat (oxidative) or 75 mM NaCl (salt) stress conditions. Fresh weight was determined by weighing a pool of 32 seedlings from each plate, with four replicates (plates) in each experiment repeated in two independent experiments (n = 8). Asterisks indicate treatments which are significant different (P<0.05) compared to the Col-0 grown on 0 mM 3 MB.

Down-regulation of PARP by RNAi has been previously shown to improve plant tolerance against short-term excessive light, heat and drought stresses [39], [44]. However, chemical PARP inhibition has only been explored in short-term experiments using highly artificial systems such as cell cultures or plant explants [27], [39], [60]. Therefore it is not clear whether chemical inhibition of PARP is suitable for improving stress tolerance in either the whole plant context or in long-term, non-lethal stress. As an inhibitor we selected 3-Methoxybenzamide (3 MB) as it is a well described PARP inhibitor in various organisms like fungi, yeast and plants [61], [62], [25] and was demonstrated already to increase stress tolerance of plant explants [39]. To obtain the best inhibitor dosage we compared five different concentrations of 3 MB in a dose response assay including a *PARP2*::RNAi line as control for reduced PARP activity. These lines were chosen as PARP enzymatic activity has been previously demonstrated to be reduced in *PARP2*::RNAi [39] and so provide a good control for these experiments. The optimal concentration was 0.2 mM 3 MB, resulting in similar growth in our plate based assay as observed for the line where PARP activity was genetically down-regulated by RNAi (Fig. 1A). Notable is that in these lines no significant enhancement was found by chemical PARP inhibition. To further investigate if chemical PARP inhibition is a suitable tool for our analysis we tested the response of plants to conditions where PARP knock-down by RNAi was previously reported to lead to enhanced tolerance, such as short-term heat and excess of light. In addition, we studied long-term oxidative (Paraquat) and salt stress. In both short-term stress assays, similar to the effect previously shown for plants with PARP RNAi, plants grown with 3 MB showed significantly enhanced biomass production, whilst the control plants only had a small and insignificant biomass increase (Fig. 1B). In the long-term assay there was a significant biomass increase of approximately 20% for control plants due to the addition of PARP inhibitor (Fig. 1B). This was consistent with the dose-response assay (Fig. 1A). However, the effect was more pronounced in both short- and long-term stress conditions, where it increased biomass up to 40% of the respective controls due to the addition of 3 MB (Fig. 1B). In summary, we demonstrated that chemical PARP inhibition could enhance whole plant growth under control conditions and in short- and long-term stresses.

### Chemical PARP inhibition alters gene expression and metabolite levels under non-stress conditions

**Figure 2:**
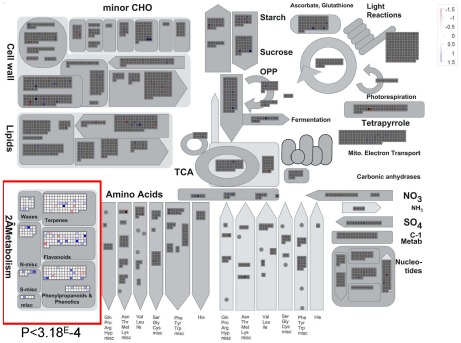
Transcriptional changes induced by PARP inhibition displayed on MAPMAN. Arabidopsis plants were grown for 18 days at 80–100 µE, 22°C on half MS medium in the presence (+3 MB) or absence (−3 MB) of 0.2 mM of the PARP inhibitor 3-Methoxy-benzamide (3 MB) within the media. For the expression analysis four samples from four different plates were pooled for both conditions in each experiment and frozen immediately in liquid nitrogen, repeated in two independent experiments. The RNA was extracted according to the requirements of the Affymetrix-microarray facility at the VIB (MAF) and processed according to the manufactures instructions.

The enhanced growth and stress tolerance conferred by chemical PARP inhibition led us to investigate whether these phenotypes were accompanied or could be explained by underlying molecular changes. Therefore, we performed both gene expression and metabolite profiling on plants grown under control conditions in the presence and absence of the chemical PARP inhibitor 3 MB. To identify responsive biochemical pathways or functional groups, the results of the expression analysis were analyzed using MAPMAN [59], and plotted on the metabolism overview diagram. Statistical analysis with the Wilcoxon rank sum test showed an enrichment of secondary metabolism genes (Fig. 2). One prominent group of changes was associated with flavonoid metabolism in general and in particular anthocyanin biosynthesis, one of the branches of the phenyl-propanoid pathway. The identification of secondary metabolism as an interesting avenue for further study was additionally supported by the GC-MS metabolite profiling, which showed reduced sinapic acid content in plants with inhibited PARP activity. In addition, the metabolite analysis also showed reduced levels of other stress responsive molecules, such as galactinol or myo-inositol (Table 1). Based on these data we hypothesized that PARP deregulation influences the phenyl-propanoid pathway. This hypothesis was further supported by observations during the long-term oxidative stress treatment, in which we found that plants grown with the PARP inhibitor 3 MB showed a green leaf color, while those without displayed brown/purple leaf coloration. Therefore we setup further experiments which allowed us to test the effect of PARP inhibition on plant pigmentation and the accumulation of anthocyanin. We used two described [19], [63] anthocyanin inductive conditions, composed of photochemical reactive oxygen production (Paraquat) and exogenous sucrose stress. In both stress conditions PARP inhibition led to enhanced growth of the plants (Figure S1) and reduced brown leaf coloration. In summary, we showed that chemical PARP inhibition is altering the expression of genes related to secondary metabolism, affects the accumulation of stress related metabolites and changes the leaf coloration under stress conditions. Nevertheless, plants growing under these conditions displayed an enhanced growth.

**Table 1:** Metabolite changes induced by PARP inhibition and detected by GC-MS.

Metabolite	Fold-change (+3 MB)
Arabinose	1.78
Ethanolamin	0.82
Fumarate	0.46
Galactinol	0.58
Galactonic acid	0.82
Myo-inositol	0.65
Sinapic acid	0.45

For the GC-MS metabolite profiling samples, a total of ∼10 seedlings from 4 plates were harvested individually for both conditions (+/−3 MB) in each experiment, repeated in three individual experiments (n = 12). The plants were grown in parallel to those for the microarray expression analysis. The metabolite extraction and further processing was done at the “Plateforme Métabolisme-Métabolome (IFR87)” accordingly to (55). Metabolites that were significantly different (*t*-test, p<0.05, Bonferroni-Holm multiple testing correction) between the 3 MB treated plants (+3 MB) and the control plants (−3 MB) are shown. Values presented are the ratio (fold-change) in the abundance of +3 MB relative to −3 MB.

### PARP inhibition reduces anthocyanin accumulation in stress conditions

**Figure 3:**
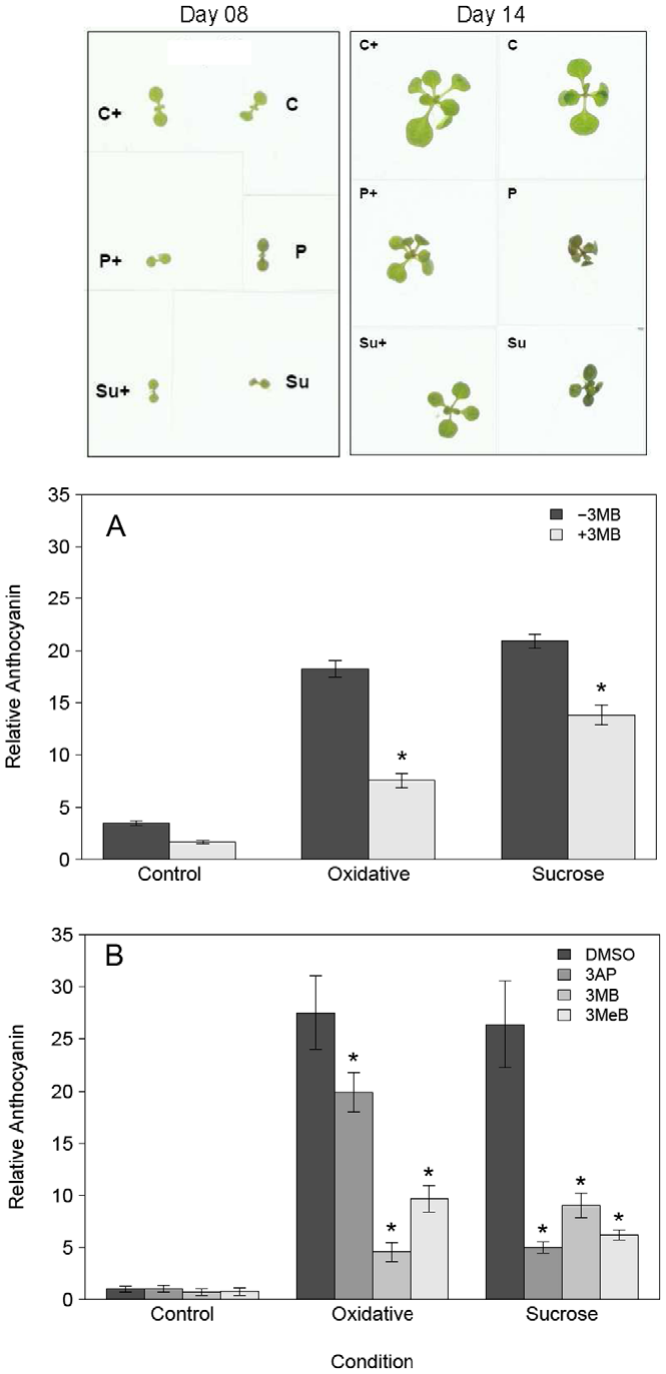
PARP inhibition reduces anthocyanin accumulation under stress. *Arabidopsis* plants (Col-0) were grown at 80–100 µE, 22°C on MS medium with (+3 MB) or without (−3 MB) the PARP inhibitor 3-Methoxy-benzamide (3 MB) and subjected to three different conditions: control, oxidative stress (0.1 µM Paraquat) or sucrose stress (150 mM Sucrose) and harvested after 14 days. A, indicates the whole plant phenotype of representative plants from all treatments. B, indicates the relative anthocyanin content after 14 days. Data are combined from three independent experiments with 5 replicates in each experiment (n = 15). C, shows the effect of different PARP inhibitors used: 3-Methoxy-benzamide (3 MB), 3-Methyl-benzamide (3MeB) and 3-Aminophthalaminhydrazide (3AP). The relative anthocyanin content is shown, combined from two independent experiments with 5 replicates in each experiment (n = 10). Asterisks indicate significant differences (P<0.05) of plants grown with 3 MB compared to those without 3 MB in the same condition.

As mentioned in the previous section, we noted that PARP inhibited seedlings stayed green, while those grown under stress without 3 MB displayed a brown/purple leaf coloration (Fig. 3). The strongest difference was observed at 14 day old plants, therefore further analysis is focusing on plants of this age. We assumed that the most likely candidate for this change in color is a difference in the accumulation of anthocyanin, which has been documented to increase under diverse stress conditions [22], [12]. We measured relative anthocyanin content in shoots of plants grown with high exogenous sucrose or oxidative stress. These experiments showed that the changed coloration was mainly due to differential anthocyanin accumulation in response to PARP inhibition (Fig. 3A). Chemical PARP inhibition led to a strong reduction in anthocyanin content, between 60% to 75% for sucrose stress and oxidative stress, respectively. In order to determine if this effect was a general effect of chemical PARP inhibition rather than specific for 3 MB, we tested two other chemical PARP inhibitors, 3-Methylbenzamide (3MeB) and 3-Aminophthalhydrazide (3AP) [62] and found similar responses (Fig. 3B). In summary, these data show that PARP inhibition reduces stress-induced anthocyanin accumulation in various inductive conditions and at different growth stages.

### PARP affects the anthocyanin biosynthesis pathway at the transcriptional level

Despite some conflicting reports [64], [44], this and previous studies have indicated that PARP inhibition can affect flavonoid metabolism. For example, PARP inhibition with 3-Aminobenzamide (3AB) reduced Phenylalanine-ammonium-lyase (PAL) enzyme activity induced in stressed *C.roses* cell cultures (64) and in biotic elicitor experiments in *Arabidopsis* [65]. In contrast, the only reported PARP mediated effect on the transcriptional level was the induction of the MYB transcription factor PAP1, a strong positive regulator of the anthocyanin biosynthesis pathway [19], in *PARP2*::RNAi lines following 6 h of light stress [44]. However, neither study investigated the whole pathway in more detail or with combined transcriptional and biochemical analysis.

**Figure 4:**
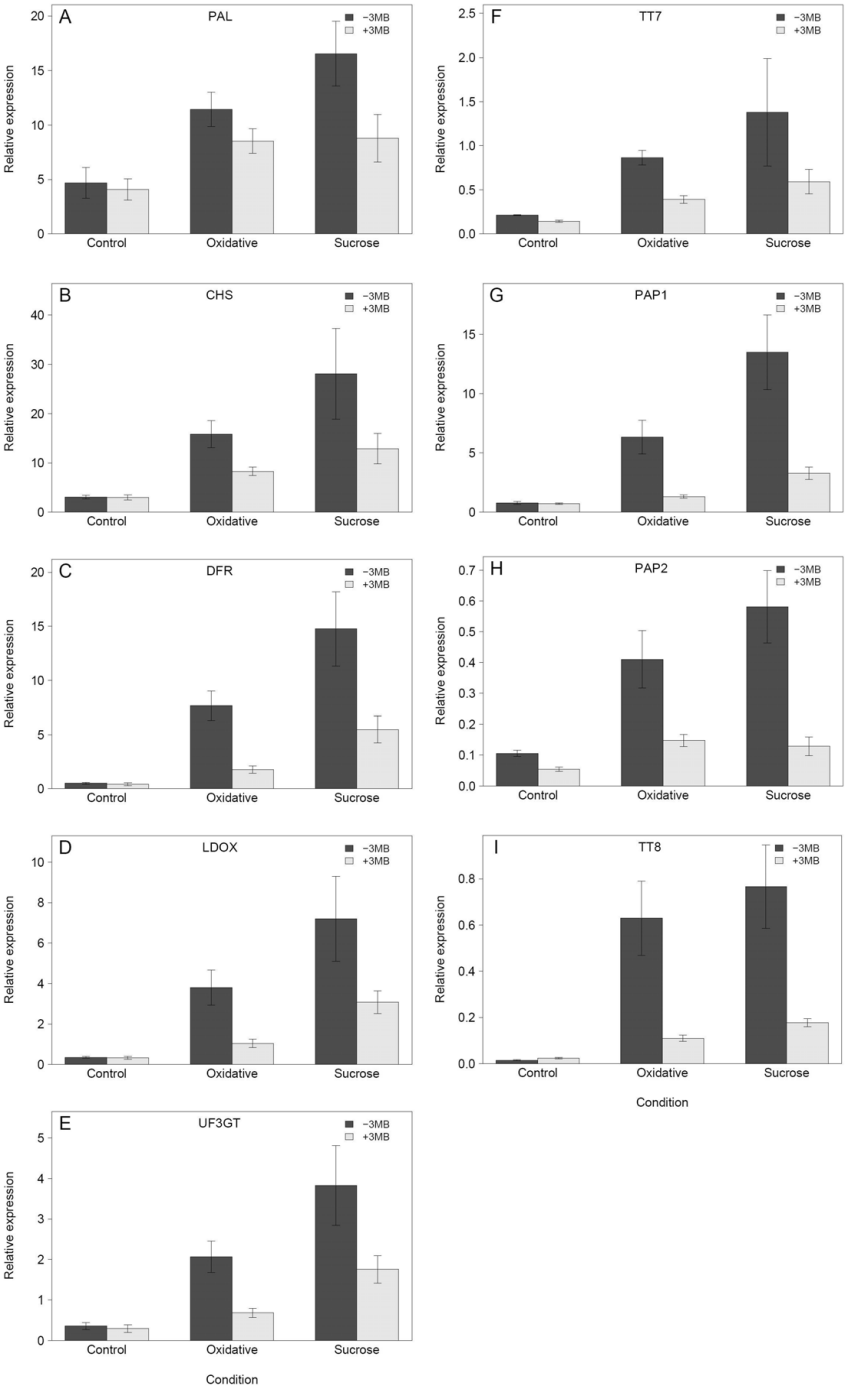
PARP inhibition reduces the transcriptional induction of anthocyanin pathway genes. *Arabidopsis* plants (Col-0) were grown at 80–100 µE, 22°C on MS medium with (+3 MB) or without (−3 MB) the PARP inhibitor 3-Methoxy-benzamide (3 MB) and subjected to three different conditions: control, oxidative stress (0.1 µM Paraquat) or sucrose stress (150 mM Sucrose) and harvested after 14 days. RNA was extracted from seedlings pooled from all five replicates within one experiment, with three independent experiments (n = 3). The average relative expression, normalized against the housekeeping gene *PP2A*, is shown, for the biosynthetic (A–F) and the regulatory (G–I) genes.

**Figure 5:**
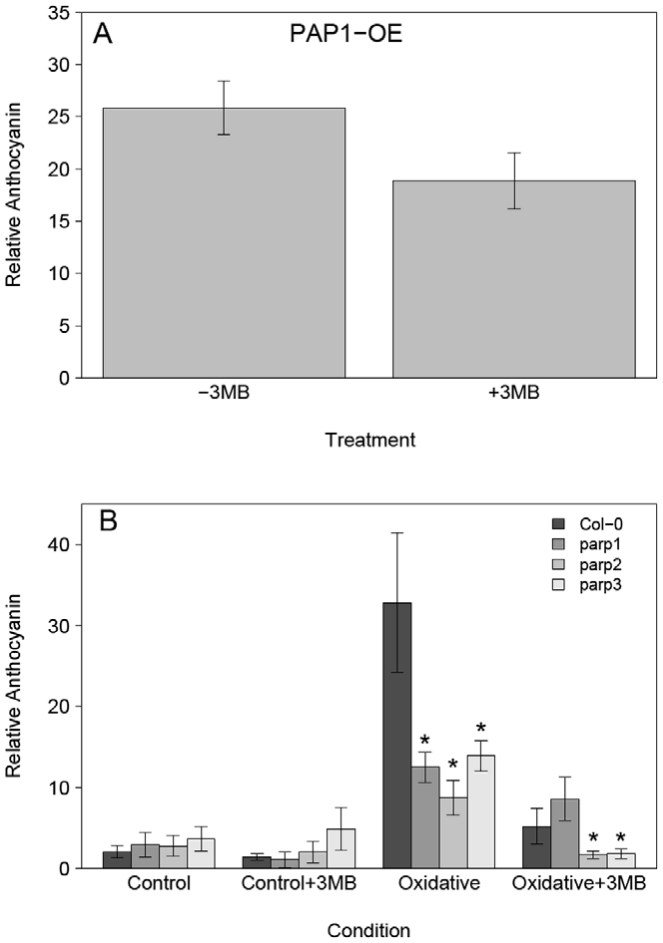
PARP inhibitory effect on anthocyanin accumulation is associated genetically with PARP activity and based on transcriptional control. The *Col-0*, *PAP1-OX* and T-DNA insertion lines of the three *PARP* genes (*parp1*, *parp2*, *parp3*) were grown for 14 days at 80–100 µE, 22°C on MS medium with (+3 MB) or without (−3 MB) 0.2 mM of the PARP inhibitor 3-Methoxy-benzamide (3 MB) in the medium. A, the relative anthocyanin content of the *PAP1*-OX line is shown, combined from three independent experiments with three replicates in each experiment (n = 9). The reduction of ∼20% is not significant (P = 0.07) and is much smaller than the reductions after 14 days for oxidative (∼70%) or sucrose (∼50%) (see Fig. 3B). B, the mutant lines were subjected to two different conditions; control or oxidative stress (0.1 µM Paraquat). The relative anthocyanin content is shown. Data are combined from 3 independent experiments with two to four replicate plates in each experiment (n = 9). Asterisks indicate significant difference (P<0.05) of the mutant lines compare to the Col-0 grown in the same condition.

As it has been previously demonstrated that anthocyanin biosynthesis is mainly controlled transcriptional [12], [19], we assessed the transcript levels of key regulatory transcription factors and biosynthetic enzymes such as *PAP1*, *TT8* and *CHS* to establish the underlying basis of the reduced anthocyanin accumulation. These analyses demonstrated that PARP inhibition reduces the expression of biosynthetic and regulatory genes under stress and that this effect was strongest for oxidative stress (Fig. 4). Thus, the down-regulation of these genes is correlated with the different reduction in anthocyanin accumulation in oxidative and sucrose stress. Also, although the effect was less pronounced in sucrose stress, it nevertheless showed the same general effect of PARP inhibition. These data suggested that the PARP effect on anthocyanin accumulation was based mainly on transcription and is not due to a reduced enzymatic activity. To test this we selected a *Production of Anthocyanin Pigment 1* over-expression line (35S::*PAP1*) and measured the relative anthocyanin content. In this background it should be possible to overcome the transcriptional repression of the pathway. The result showed that PARP inhibition was not able to reduce the relative anthocyanin content significantly (Fig. 5A), supporting the idea that the effect is based on transcriptional regulation. To confirm that the effect of reduced PARP activity on the anthocyanin accumulation was not specific to chemical inhibition, we used homozygous T-DNA knock-out lines for all three *PARP* genes. The experiments showed that all the *parp* knock-outs have a similarly reduced anthocyanin accumulation under stress but that this reduction is not as strong as with chemical inhibition (Fig. 5B). This might indicate that the reduction in anthocyanin is caused by inhibition of multiple isoforms. Noteworthy is that chemical inhibition in the *parp2* and *parp3* background under stress lead to a further reduced anthocyanin accumulation, but not in the parp1 background. In summary these results indicate that PARP inhibition influences anthocyanin accumulation at the transcriptional level and that the effect was strongest for the major regulatory genes which may be responsible for the observed down-regulation of the later steps of the biosynthetic pathway.

### PARP inhibition reduces anthocyanin as well as other defensive molecule accumulation without photosynthetic penalty

**Figure 6:**
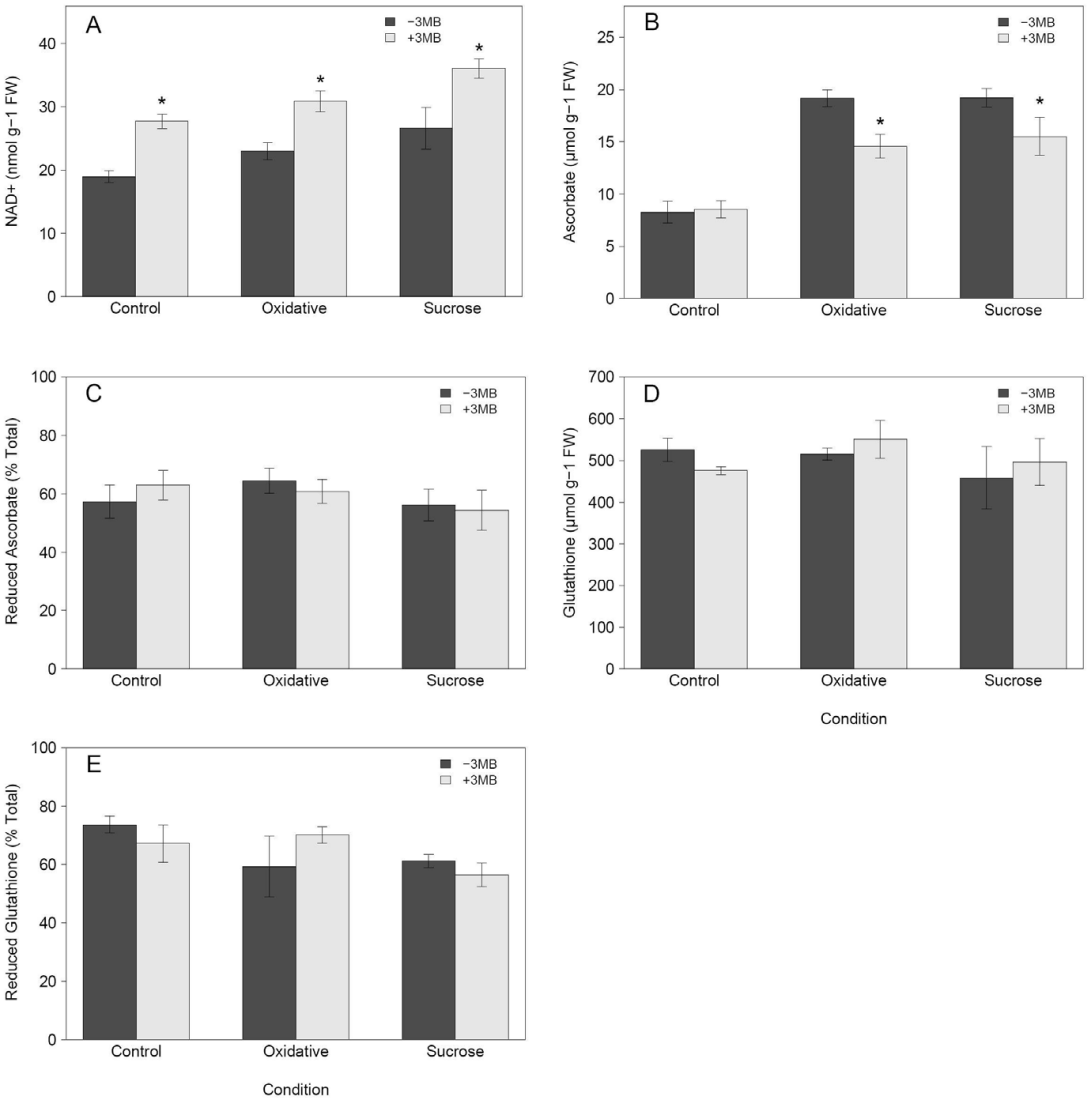
Chemical PARP inhibition changes cellular redox profiles. Arabidopsis Col-0 seedlings were grown for 14 days at 80–100 µE, 22°C on MS media with (+3 MB) or without (−3 MB) the PARP inhibitor 3-Methoxy-benzamide (3 MB) and were subjected to three different treatments: control, oxidative stress (0.1 µM Paraquat) or sucrose stress (150 mM sucrose). Shown are (A) the NAD+ content, (B) the total ascorbate content, (C) the reduction level of the ascorbate, (D) the total glutathione and (E) the reduction of the total glutathione. Data are combined from three independent experiments with 2 replicates in each experiment (n = 6). Asterisks indicate significant difference (P<0.05) compared to Col-0 grown in the same condition without 3 MB.

**Figure 7:**
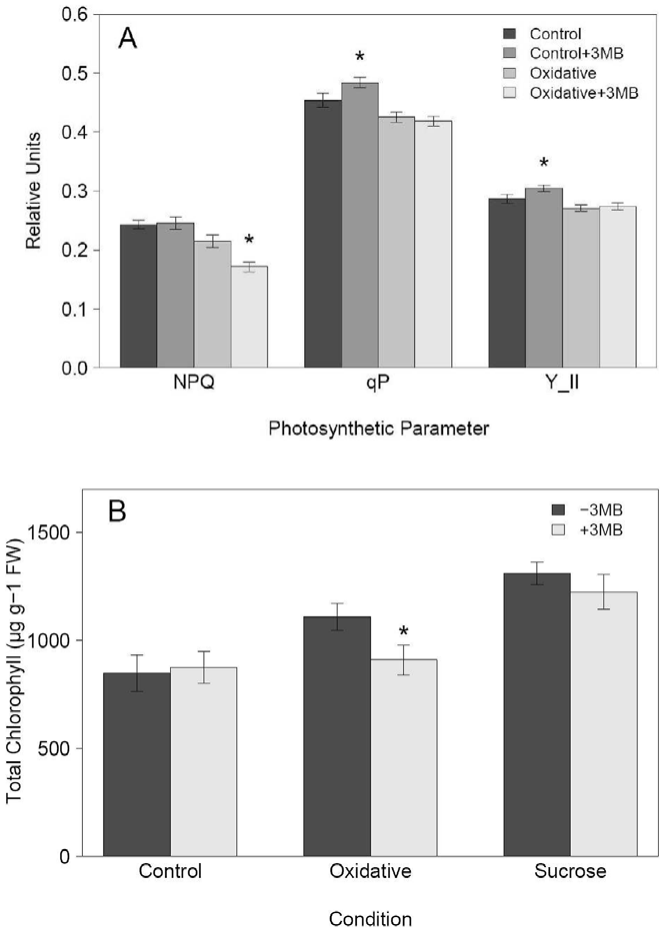
Chemical PARP inhibition alters photosynthesis. *Arabidopsis* seedlings (Col-0) were grown at 80–100 µE, 22°C on MS medium with (+3 MB) or without (−3 MB) the PARP inhibitor 3-Methoxy-benzamide (3 MB) and subjected to different conditions: control and oxidative stress (0.1 µM Paraquat) and for the chlorophyll measurements also sucrose (3.5% sucrose). A, shows the non-photochemical quenching (NPQ), the proportion of open PSII reaction centers (qP) and the effective quantum yield (Y_II) measured via PAM imaging. B, shows the chlorophyll content after 8 days. For (A) 8–18 seedlings were analyzed in each of the 4 independent experiments (n = 42); for (B) data are combined from three independent experiments with 5 replicates in each experiment (n = 15). Asterisks indicate significant difference (P<0.05) between seedlings grown in the same condition but treated with 3 MB or without 3 MB.

Previous investigations of PARP inhibition have shown links with energy metabolism and cellular redox status. For example, RNAi down-regulation of PARP leads to an increased NAD+ content in stress conditions [39], and cell-culture studies have provided support for a link between PARP activity and glutathione content [50]. To investigate the effects on redox metabolism of chemical PARP inhibition in whole plants subjected to long term stress, we performed a detailed redox-profiling. This analysis revealed that the expected increase in NAD+ content was also evident following chemical PARP inhibition, leading to an increase over time of about 30% in NAD+ content (Fig. 6A) in control and stress conditions. Despite this change in NAD+, the other closely related metabolites NADH, NADP(H) did not change significantly (data not shown). Glutathione did not show notable changes, neither in the amount nor in the reduction status (Fig. 6D, E). More importantly, we observed a significant decrease of more than 20% in the total ascorbate content due to chemical inhibition of PARP activity for both sucrose and oxidative stress (Fig. 6B, C) coinciding with the observed decrease in anthocyanin accumulation. In addition, drought protective molecules such as galactinol accumulate less in PARP inhibited plants (Table 1). Taking into account the necessity of water availability and redox balance for photosynthetic performance, the reduced antioxidant capacity due to reduced anthocyanin and ascorbate content as well as previous evidence of increased chlorophyll content in *PARP2*::RNAi lines [39], we decided to perform a detailed photosynthetic analysis via PAM imaging and chlorophyll measurements. To asses early effects potentially leading to the enhanced growth and increased stress tolerance we used 8 day instead of 14 day old plants for the chlorophyll fluorescence (PAM) measurement. At day 8 it was also possible to measured differences in the relative anthocyanin content (Figure S2). No negative impact of the reduced anthocyanin accumulation was found by PAM imaging under stress conditions. On the contrary, weak but significant positive effects were found on the effective quantum yield (Φ_PSII_), the proportion of open PSII reaction centers whilst non-photochemical quenching (NPQ) was reduced (Fig. 7A). In contrast to previous results obtained in *PARP2*::RNAi lines in a drought experiment including a re-hydration step [39], we found that PARP inhibition showed no effect on chlorophyll content under control and sucrose stress, but led to a weak reduction under oxidative stress (Fig. 7B). Interestingly, at day 8 we did not observe any significant changes in the redox profiles (Figure S3).

In summary, these experiments showed that next to reduced anthocyanin content, chemical PARP inhibition also leads to a reduced induction of ascorbate under stress. The reduced accumulation of these two defense molecules has no negative effect on photosynthetic performance of the plants under the conditions studied. Furthermore, we demonstrated that chemical PARP inhibition is able to enhance NAD+ content.

## Discussion

### Effect of chemical PARP inhibition in whole plants

In plant science chemical genetics is often used to overcome genetic redundancy [66], [67], [68] and was successfully applied in recent years to elucidate e.g. the ABA receptor family or the influence of glycogen synthase kinase 3 like kinases (GSK3-like) in brassinosteroid signaling [69], [70]. The use of chemicals to increase abiotic stress tolerance has been reported, for example using plant hormones such as ABA, ethylene, brassinosteroids or through molecules of undefined action that prime defense pathways [71], [72], [73], [74]. However, chemical approaches to increase growth and tolerance of plants to abiotic stress by targeting specific enzymes or acting via established inhibitory modes of action have received less attention. The first goal of this study was to test if chemical PARP inhibition is applicable to whole plants and able to increase abiotic stress tolerance in a similar way as previously shown for genetic PARP inhibition. Chemical PARP inhibitors have been previously used, but often within non-physiological concentration ranges, in plant cell cultures or in short-term assays following transfer to media containing the inhibitor [27], [39], [75], [65]. Our long-term experiments showed that inhibitor concentrations typically used for short-term experiments result in severe growth reduction (Fig. 1), whereas an appropriate lower inhibitor concentration allows successful recapitulation of the positive genetic down-regulation effects in heat and high-light stress, as previously reported [39], [44]. Contrary to the severe short-term abiotic stress treatments [76], [77], [78], we focused on the response to a mild and long-term stress. These have been recently highlighted as being more valuable method for detailed analysis of growth responses under stress [79], [80]. Our data show that chemically inhibited PARP activity leads to increased plant growth during long- and short-term stress events. It should be noted that PARP inhibition also increased growth under the control conditions used in our study. As these conditions are typical of those employed in plate-based assays we thus consider them to be representative for control conditions. However, we cannot exclude that to some limited degree they also represent a stress condition because: 1) the redox balance ratio is much lower in comparison to soil grown plants [81]; 2) plate grown plants may show hypoxia responses as indicated by enhanced alcohol dehydrogenase activity [82]. Overall, we demonstrate that chemical enzyme activity modulation, especially chemical PARP inhibition 1) recapitulates genetic phenotypes; 2) is applicable to whole plants and 3) could enhance growth under control conditions as well as tolerance and growth under long-term stress. This opens further perspectives for the use of new chemicals to enhance crop abiotic stress tolerance and productivity.

### PARP inhibition transcriptional regulates anthocyanin accumulation

In our experiments it was clearly observed that chemical PARP inhibition allows the stressed plants to stay green in a similar way to control plants and not change color to dark green/brown as observed for those grown under stress without PARP inhibition. We demonstrated that this was due to reduced anthocyanin accumulation in response to oxidative and sucrose stress conditions [12], [22]. To confirm our data showing reduced anthocyanin accumulation we used PARP inhibitors of different chemical classes [62] to avoid that the inhibitors bind to similar artificial targets. The unspecific binding of PARP inhibitors to mono-ADP transferases is a general problem, but the mainly used 3-methoxybenzamide inhibitor showed a 1000× lower affinity to mono-ADP transferases compared to poly(ADP)ribose polymerases [62], [83]. This combined with the used concentration makes it likely that this is not a relevant factor within the experiments.

In addition, we demonstrated that T-DNA mutant lines of the three *PARP* genes also showed a significant reduction in anthocyanin accumulation under stress, while keeping in account that the PARP protein family is partly redundant in their effect on anthocyanin accumulation and on the other hand that chemical PARP inhibition allows targeting of all isoforms.

The microarray expression profiling indicated a strong impact of PARP inhibition on secondary metabolism. This was later confirmed by targeted qRT-PCR of anthocyanin regulatory and biosynthesis genes. Repressed transcription likely leads to the observed reduction in anthocyanin accumulation. The strongest transcriptional effect was observed for enzymes catalyzing the last steps in the anthocyanin biosynthesis pathway and for two key regulatory genes, *PAP1* and *TT8*, indicating that these genes are likely responsible for the reduced anthocyanin content. Alternatively, this effect can also be a result of a reduction of PAL activity, as a *pal1pal2* double mutant showed reduced anthocyanin accumulation [84], [85] and PAL activity was reduced in cell cultures under stress following application of a chemical PARP inhibitor, albeit at very high inhibitor doses (3-Aminobenzamide) [64]. However, we do not favor this alternative because the previously demonstrated reduced PAL activity did not lead to altered anthocyanin content [64] and we did not observe any significant affect of PARP inhibition on the expression of *PAL1* which encodes the main isoform of the four partly redundant PAL enzymes. Based on these data we favor the hypothesis that PARP influences anthocyanin accumulation at the transcriptional rather than post-transcriptional level. To further exclude the possibility for post-transcriptional regulation, we tested whether PARP inhibition could reduce anthocyanin biosynthesis in the 35S::*PAP1* line, which accumulates anthocyanin due to transcriptional activation of the pathway via the PAP1 transcription factor [19]. There was only a small, non-significant reduction of the anthocyanin content in the 35S::*PAP1* plants, which is in line with the hypothesis that PARP most likely influences anthocyanin accumulation at the transcriptional level and not by post-transcriptional regulation. This finding is partially contradictory to previous results obtained in *PARP2*::RNAi lines, where under light stress *PAP1*, *DFR* and *LDOX* were induced [44]. However, this seems most likely to be related to the different experimental conditions, short-term severe high light versus long-term moderate oxidative and sucrose stress.

Although the effect of repressed anthocyanin accumulation was seen in both stresses, we noted that chemical PARP inhibition reduced anthocyanin accumulation to a greater degree in oxidative than in sucrose stress. This might be due to a hormonal effect on anthocyanin accumulation as shown before [86], [87], especially as ABA was shown to induce the production of anthocyanin in plants grown on sucrose [21]. Plants with reduced PARP activity also have an elevated ABA content [44]. A possible explanation can be the differential involvement or activation of the ABA pathway during oxidative and sucrose stress conditions, hence explaining the smaller reduction of anthocyanin accumulation by PARP inhibition under sucrose stress.

### PARP inhibition increases NAD+ content, improves photosynthesis and growth under stress

In our long-term stress treatments, no NAD+ depletion was found in comparison to control conditions, contrary to studies using short-term treatments [39]. We also observed no lesions on the leaves as indicators for necrotic cell death, a process often associated with a depletion of NAD+ or ATP [27], [88], [39], [89]. Availability and recycling of NAD+ are crucial processes to maintain energy homeostasis and both are influenced by PARP activity. Our results demonstrate that, in addition to genetic down-regulation [39], [44] a chemical approach of reducing PARP activity could also improve abiotic stress tolerance in whole plants. The chemically reduced PARP activity leads to increased energy levels, indicated by the increased NAD+ content in control and both stress conditions. At the same time, reduced PARP activity leads to a reduced accumulation of protective molecules, especially anthocyanin and ascorbate but also others like myo-inositol, which could be also an indirect effect via altered energy signaling [90]. Our results illustrate that reduced PARP activity is correlated with reduced ascorbate accumulation in stress, providing a link between PARP activity and redox processes under stress, in addition to the correlation with glutathione proposed before for developmental processes [50], [91]. In our experiments, we showed an overall increased NAD+ content and showed that photosynthesis is positively affected by PARP inhibition, supporting a close link between photosynthesis and PARP as hypothesized in [45]. This might be based on the altered NAD+ content, but could also be a consequence of altered protein turnover or activity influenced by PARP itself. It is known that photosynthesis, energy homeostasis, redox balance and metabolism are closely related [92] and our study provides evidence that PARP activity is linked to all of them. This supports a model where PARP is a central regulator in plant stress tolerance at the cellular and whole plant level as suggested by [44]. The interaction of PARP with NAD+ and its effect on the redox balance and photosynthesis leads to growth enhancement associated with a reduced induction of protective pathways. Whether the observed reduced defense will be present in field conditions remains elusive. Anthocyanin is associated with stress tolerance in e.g. winter-green plants, particular in resistance to drought but is not a pre-requisite for successful adaptation [93]. Higher ascorbate content might be beneficial in stress situations but a lack could be compensated by higher presence of other small molecules such as α-Tocopherol [94]. In summary, our study provided an elaborate physiological analysis to demonstrate that chemical PARP inhibition represses the accumulation of defensive molecules, especially of anthocyanin. We also showed that it is possible to recapitulate the effect of genetically reduced PARP activity in a whole plant context using chemical inhibition. Furthermore, the results demonstrate that chemical PARP inhibition increased tolerance against very different stress conditions such as oxidative and sucrose stress. Biochemical and genetic analysis revealed a reduced accumulation of stress protection molecules such as ascorbate, galactinol and anthocyanins. Reduction of anthocyanin accumulation is mainly controlled at the transcriptional level. In addition, our data supports a link between PARP activity with redox balance and photosynthesis. Consequently, these changes led to enhanced growth under control and stress conditions.

## Supporting Information

Figure S1 **PARP inhibition leads to enhanced tolerance against long-term stress.** Plants were grown for 14 days at 80–100 µE, 22°C on MS medium and subjected to five different conditions: control, oxidative stress (0.1 µM paraquat), sucrose stress (150 mM sucrose), osmotic stress (100 mM sorbitol) and salt stress (75 mM NaCl). The average fresh weight of individual plants was determined by weighing 32 pooled seedlings from each plate, with 5 replicates (plates) in each experiment repeated in three independent experiments (n = 15). Significant differences (P<0.05) between the seedlings grown with PARP inhibitor compared to those grown without in the same condition is indicated by an asterisk.
(TIF)

Figure S2 **Chemically PARP inhibition reduces anthocyanin accumulation already at early stages.** Arabidopsis seedlings (Col-0) were grown for 8 days at 80–100 µE, 22°C on MS medium and subjected to three conditions: control, oxidative stress (0.1 µM paraquat) or sucrose stress (150 mM sucrose) with (+3 MB) or without (−3 MB) the PARP inhibitor 3-Methoxy-benzamide in the media. The relative anthocyanin content is shown, data are combined from three independent experiments with 5 replicates in each experiment (n = 15). Asterisks indicate significant difference (P<0.05) in anthocyanin accumulation between seedlings grown with a PARP inhibitor compare to those without in the same condition.
(TIF)

Figure S3 **The effect of chemical PARP inhibition on cellular redox profiles at 8 days.** *Arabidopsis* Col-0 seedlings were grown for 8 days at 80–100 µE, 22°C on MS media with (+3 MB) or without (−3 MB) the PARP inhibitor 3-Methoxy-benzamide (3 MB) and were subjected to three different treatments: control, oxidative stress (0.1 µM Paraquat) or sucrose stress (150 mM sucrose). Shown are (A) the NAD+ content, (B) the total ascorbate content, (C) the reduction level of the ascorbate, (D) the total glutathione and (E) the reduction of the total glutathione. Data are combined from three independent experiments with 1 or 2 replicates in each experiment (n = 4). Asterisks indicate significant difference (P<0.05) compared to Col-0 grown in the same condition without 3 MB.
(TIF)

## References

[1] Lippold F, Sanchez DH, Musialak M, Schlereth A, Scheible WR (2009). AtMyb41 regulates transcriptional and metabolic responses to osmotic stress in Arabidopsis.. Plant Physiol.

[2] Larkindale J, Vierling E (2008). Core genome responses involved in acclimation to high temperature.. Plant Physiol.

[3] Mittler R, Vanderauwera S, Gollery M, Van Breusegem F (2004). Reactive oxygen gene network of plants.. Trends Plant Sci.

[4] Seki M, Umezawa T, Urano K, Shinozaki K (2007). Regulatory metabolic networks in drought stress responses.. Curr Opin Plant Biol.

[5] Miller G, Suzuki N, Ciftci-Yilmaz S, Mittler R (2010). Reactive oxygen species homeostasis and signaling during drought and salinity stress.. Plant cell and Enviro.

[6] Rizhsky L, Davletova S, Liang H, Mittler R (2004). The zinc finger protein Zat12 is required for cytosolic ascorbate peroxidase 1 expression during oxidative stress in Arabidopsis.. J Biol Chem.

[7] Rossel JB, Walter PB, Hendrickson L, Chow WS, Poole A (2006). A mutation affecting ASCORBATE PEROXIDASE 2 gene expression reveals a link between responses to high light and drought tolerance.. Plant Cell Environ.

[8] Oh SJ, Song SI, Kim YS, Jang HJ, Kim SY (2005). Arabidopsis CBF3/DREB1A and ABF3 in transgenic rice increased tolerance to abiotic stress without stunting growth.. Plant Physiol.

[9] Nelson DE, Repetti PP, Adams TR, Creelman RA, Wu J (2007). Plant nuclear factor Y (NF-Y) B subunits confer drought tolerance and lead to improved corn yields on water-limited acres.. Proc Natl Acad Sci USA.

[10] Rossel JB, Wilson IW, Pogson BJ (2002). Global changes in gene expression in response to high light in Arabidopsis.. Plant Physiol.

[11] Hernández I, Van Breusegem F (2010). Opinion on the possible role of flavonoids as energy escape valves: Novel tools for nature's Swiss army knife?. Plant Sci.

[12] Vanderauwera S, Zimmermann P, Rombauts S, Vandenabeele S, Langebartels C (2005). Genome-wide analysis of hydrogen peroxide-regulated gene expression in Arabidopsis reveals a high light-induced transcriptional cluster involved in anthocyanin biosynthesis.. Plant Physiol.

[13] Cominelli E, Gusmaroli G, Allegra D, Galbiati M, Wade HK (2008). Expression analysis of anthocyanin regulatory genes in response to different light qualities in Arabidopsis thaliana.. J Plant Physiol.

[14] Gould KS, Vogelmann TC, Han T, Clearwater MJ (2002). Profiles of photosynthesis within red and green leaves of Quintinia serrata.. Physiol Plant.

[15] Nagata T, Todoriki S, Masumizu T, Suda I, Furuta S (2003). Levels of active oxygen species are controlled by ascorbic acid and anthocyanin in Arabidopsis.. J Agri Food Chem.

[16] Hernández I, Alegre L, Van Breusegem F, Munné-Bosch S (2009). How relevant are flavonoids as antioxidants in plants?. Trends Plant Sci.

[17] Scheible WR, Morcuende R, Czechowski T, Fritz C, Osuna D (2004). Genome-wide reprogramming of primary and secondary metabolism, protein synthesis, cellular growth processes, and the regulatory infrastructure of Arabidopsis in response to nitrogen.. Plant Physiol.

[18] Morcuende R, Bari R, Gibon Y, Zheng W, Pant BD (2007). Genome-wide reprogramming of metabolism and regulatory networks of Arabidopsis in response to phosphorus.. Plant Cell Environ.

[19] Tohge T, Nishiyama Y, Hirai MY, Yano M, Nakajima J (2005). Functional genomics by integrated analysis of metabolome and transcriptome of Arabidopsis plants over-expressing an MYB transcription factor.. Plant J.

[20] Vandenbussche F, Habricot Y, Condiff AS, Maldiney R, Van der Straeten D (2007). HY5 is a point of convergence between cryptochrome and cytokinin signalling pathways in Arabidopsis thaliana.. Plant J.

[21] Loreti E, Povero G, Novi G, Solfanelli C, Alpi A (2008). Gibberellins, jasmonate and abscisic acid modulate the sucrose-induced expression of anthocyanin biosynthetic genes in Arabidopsis.. New Phytol.

[22] Teng S, Keurentjes J, Bentsink L, Koornneef M, Smeekens J (2005). Sucrose-specific induction of anthocyanin biosynthesis in Arabidopsis requires the MYB75/PAP1 gene.. Plant Physiol.

[23] Baudry A, Caboche M, Lepiniec L (2006). TT8 controls its own expression in a feedback regulation involving TTG1 and homologous MYB and bHLH factors, allowing a strong and cell-specific accumulation of flavonoids in Arabidopsis thaliana.. Plant J.

[24] Dubos C, Le Gourrierec J, Baudry A, Huep G, Lanet E (2008). MYBL2 is a new regulator of flavonoid biosynthesis in Arabidopsis thaliana.. Plant J.

[25] Chen YM, Shall S, O'Farrell M (1994). Poly(ADP-ribose) polymerase in plant nuclei.. Eur J Biochem.

[26] Lepiniec L, Babiychuk E, Kushnir S, Van Montagu M, Inzé D (1995). Characterization of an Arabidopsis thaliana cDNA homologue to animal poly(ADP-ribose) polymerase.. FEBS Lett.

[27] Amor Y, Babiychuk E, Inzé D, Levine A (1998). The involvement of poly(ADP-ribose) polymerase in the oxidative stress responses in plants.. FEBS Lett.

[28] Ruf A, de Murcia JM, de Murcia G, Schulz GE (1996). Structure of the catalytic fragment of poly(AD-ribose) polymerase from chicken.. Proc Natl Acad Sci USA.

[29] Oliver AW, Amé J, Roe SM, Good V, de Murcia G (2004). Crystal structure of the catalytic fragment of murine poly(ADP-ribose) polymerase-2.. Nucleic Acids Res.

[30] Altmeyer M, Messner S, Hassa PO, Fey M, Hottiger MO (2009). Molecular mechanism of poly(ADP-ribosyl)ation by PARP1 and identification of lysine residues as ADP-ribose acceptor sites.. Nucleic Acids Res.

[31] Briggs AG, Bent AF (2011). Poly(ADP-ribosyl)ation in plants.. TIPS.

[32] Lamb RS, Citarelli M, Teotia S (2011). Functions of the poly(ADP-ribose)polymerase superfamily in Plants.. Cell Mol Life Sci.

[33] Amé J, Spenlehauer C, de Murcia G (2004). The PARP superfamily.. BioEssays.

[34] Kim MY, Zhang T, Kraus WL (2005). Poly(ADP-ribosyl)ation by PARP-1: ‘PAR-laying’ NAD into a nuclear signal.. Genes Dev.

[35] Hassa PO, Hottiger MO (2008). The diverse biological roles of mammalian PARPS, a small but powerful family of poly-ADP-ribose polymerases.. Front Biosci.

[36] Teotia S, Lamb RS (2009). The paralogous genes RADICAL-INDUCED CELL DEATH1 and SIMILAR TO RCD ONE1 have partially redundant functions during Arabidopsis development.. Plant Physiol.

[37] Jaspers P, Overmyer K, Wrzaczek M, Vainonen JP, Blomster T (2010). The RST and PARP-like domain containing SRO protein family: analysis of protein structure, function and conservation in land plants.. BMC Genomics.

[38] Doucet-Chabeaud G, Godon C, Brutesco C, de Murcia G, Kazmaier M (2001). Ionising radiation induces the expression of PARP-1 and PARP-2 genes in Arabidopsis.. Mol Genet Genomics.

[39] De Block M, Verduyn C, De Brouwer D, Cornelissen M (2005). Poly(ADP-ribose) polymerase in plants affects energy homeostasis, cell death and stress tolerance.. Plant J.

[40] Adams-Phillips L, Wan J, Tan X, Dunning FM, Meyers BC (2008). Discovery of ADP-ribosylation and other plant defense pathway elements through expression profiling of four different Arabidopsis-Pseudomonas R-avr interactions.. Mol Plant Microbe Interact.

[41] Hunt L, Holdsworth MJ, Gray JE (2007). Nicotinamidase activity is important for germination.. Plant J.

[42] Babiychuk E, Van Montagu M, Kushnir S (2001). N-terminal domains of plant poly(ADP-ribose)polymerases define their association with mitotic chromosoms.. Plant J.

[43] Storozhenko S, Inzé D, Van Montagu M, Kushnir S (2001). Arabidopsis coactivator ALY-like proteins, DIP1 and DIP2, interact physically with the DNA-binding domain of the Zn-finger poly(ADP-ribose) polymerase.. J Exp Bot.

[44] Vanderauwera S, De Block M, Van de Steene N, van de Cotte B, Metzlaff M (2007). Silencing of poly(ADP-ribose) polymerase in plants alters abiotic stress signal transduction.. Proc Natl Acad Sci USA.

[45] Arena C, Mistretta C, Di Natale E, Faraone Mennella MR, De Santo AV (2011). Characterization and role of poly(ADP-ribosyl)ation in the Mediterranean species Cistus incanus L. under different temperature conditions.. Plant Physiol Biochem.

[46] Ha H, Snyder SH (1999). Poly(ADP-ribose) polymerase is a mediator of necrotic cell death by ATP depletion.. Proc Natl Acad Sci USA.

[47] Hashida S, Takahashi H, Uchimiya H (2009). The role of NAD biosynthesis in plant development and stress responses.. Annals of Botany.

[48] Filipovic DM, Meng X, Reeves WB (1999). Inhibition of PARP prevents oxidant-induced necrosis but not apoptosis in LLC-PK1 cells.. Am J Physiology.

[49] Virág L, Szabo C (2002). The therapeutic potential of poly(ADP-ribose) polymerase inhibitors.. Pharmacol Rev.

[50] Pellny TK, Locato V, Diaz Vivancos P, Markovic J, De Gara L (2009). Pyridine nucleotide cycling and control of intracellular redox state in relation to poly (ADP-ribose) polymerase activity and nuclear localization of glutathione during exponential growth of Arabidopsis cells in culture.. Mol Plant.

[51] Murashige T, Skoog F (1962). A Revised Medium for Rapid Growth and Bio Assays with Tobacco Tissue Cultures.. Physiol Plant.

[52] Czechowski T, Stitt M, Altman T, Udvardi MV, Scheible WR (2005). Genome-wide identification and testing of superior reference genes for transcript normalization in Arabidopsis.. Plant Physiol.

[53] Arnon D (1949). COPPER ENZYMES IN ISOLATED CHLOROPLASTS. POLYPHENOLOXIDASE IN BETA VULGARIS.. Plant Physiol.

[54] Queval G, Noctor G (2007). A plate reader method for the measurement of NAD, NADP, glutathione, and ascorbate in tissue extracts: Application to redox profiling during Arabidopsis rosette development.. Anal Biochem.

[55] Noctor G, Bergot G, Mauve C, Thominet D, Lelarge-Trouverie C (2007). A comparative study of amino acid measurements in leaf extracts by gas chromatogrphy-time of flight mass spectrometry and high performance liquid chromatography with fluorescence detection.. Metabolomics.

[56] Gentlemen RC, Carey VJ, Bates DM, Bolstad B, Dettling M (2004). Bioconductor: open software development for computational biology and bioinformatics.. Genome Biol.

[57] Gautier L, Cope L, Bolstad BM, Irizarry RA (2004). affy-analysis of Affymetrix GeneChip data at the probe level.. Bioinformatics.

[58] Smyth GK (2004). Linear models and empirical Bayes for assessing differential expression in microarray experiments.. Stat App Gen Mol Biol.

[59] Thimm O, Blasing O, Gibon Y, Nagel A, Mayer S (2004). MAPMAN: a user-driven tool to display genomics data sets onto diagrams of metabolic pathways and other biological processes.. Plant J.

[60] Tian RH, Zhang GY, Yan CH, Dai YR (2000). Involvement of poly(ADP-ribose) polymerase and activation of caspase-3-like protease in heat shock-induced apoptosis in tobacco suspension cells.. FEBS Lett.

[61] Golderer G, Gröbner P (1991). ADP-ribosylation of core histones and their acetylated subspecies.. Biochem J.

[62] Banasik M, Komura H, Shimoyama M, Ueda K (1992). Specific inhibitors of poly(ADP-ribose) synthetase and mono(ADP-ribosyl)transferase.. J Biol Chem.

[63] Solfanelli C, Poggi A, Loreti E, Alpi A, Perata P (2006). Sucrose-specific induction of the anthocyanin biosynthesis pathway in Arabidopsis.. Plant Physiol.

[64] Berglund T, Kalbin G, Strid A, Rydström J, Ohlsson AB (1996). UV-B- and oxidative stress-induced increase in nicotinamide and trigonelline and inhibition of defensive metabolism induction by poly(ADP-ribose)polymerase inhibitor in plant tissue.. FEBS Lett.

[65] Adams-Phillips L, Briggs AG, Bent AF (2010). Disruption of poly(ADP-ribosyl)ation mechanisms alters responses of Arabidopsis to biotic stress.. Plant Physiol.

[66] Cutler S, McCourt P (2005). Dude, where's my phenotype? Dealing with redundancy in signaling networks.. Plant Physiol.

[67] McCourt P, Desveaux D (2010). Plant chemical genetics.. New Phytol.

[68] Tóth R, van der Hoorn RAL (2010). Emerging principles in plant chemical genetics.. Trends Plant Sci.

[69] Park SY, Fung P, Nishimura N, Jensen DR, Fujii H (2009). Abscisic acid inhibits type 2C protein phosphatases via the PYR/PYL family of START proteins.. Science.

[70] Vert G, Chory J (2006). Downstream nuclear events in brassinosteroid signalling.. Nature.

[71] Hwang SY, Vantoai TT (1991). Absicic acid induces anaerobiosis tolerance in corn.. Plant Physiol.

[72] Clark SM, Mur LA, Wood JE, Scott IM (2004). Salicyclic acid dependent signaling promotes basal thermotolerance but is not essential for aquired thermotolerance in Arabidopsis thaliana.. Plant J.

[73] Divi UK, Rahman T, Krishna P (2010). Brassinosteroid-mediated stress tolernace in Arabidopsis thalianashows interaction with absisic acid, ethylene and salicyclic acid pathways.. BMC Plant Biol.

[74] Jakab G, Ton J, Flors V, Zimmerli L, Métraux J (2005). Enhancing Arabidopsis Salt and Drought Stress Tolerance by Chemical Priming for Its Abscisic Acid Responses.. Plant Physiol.

[75] Ishikawa T, Takahara K, Hirabayashi T, Matsumura H, Fujisawa S (2010). Metabolome analysis of response to oxidative stress in rice suspension cells overexpressing cell death suppressor Bax inhibitor-1.. Plant Cell Physiol.

[76] Miller G, Suzuki N, Rizhsky L, Hegie A, Koussevitzky S (2007). Double mutants deficient in cytosolic and thylakoid ascorbate peroxidase reveal a complex mode of interaction between reactive oxygen species, plant development, and response to abiotic stresses.. Plant Physiol.

[77] Rossel JB, Wilson PB, Hussain D, Woo NS, Gordon MJ (2007). Systemic and intracellular responses to photooxidative stress in Arabidopsis.. Plant Cell.

[78] Ge L, Chao D, Shi M, Zhu MZ, Gao J (2008). Overexpression of the trehalose-6-phosphate phosphatase gene OsTPP1 confers stress tolerance in rice and results in the activation of stress responsive genes.. Planta.

[79] Skirycz A, De Bodt S, Obata T, De Clercq I, Claeys H (2010). Developmental stage specificity and the role of mitochondrial metabolism in the response of Arabidopsis leaves to prolonged mild osmotic stress.. Plant Physiol.

[80] Skirycz A, Vandenbroucke K, Cluaw P, Maleux K, De Mayer B (2011). Survival and growth of Arabidopsis plants given limited water are not equal.. Nature Biotechnol.

[81] Queval G, Issakidis-Bourquet E, Hoeberichts FA, Vandorpe M, Gakiere B (2007). Conditional oxidative stress response in the Arabidopsis photorespiratory mutant cat2 demonstrate the redox state is a key modulator of the daylength-dependent gene expression and define photoperiode as a crucial factor in the regulation of H2O2-induced cell death.. Plant J.

[82] Chung HJ, Ferl RJ (1999). Arabidopsis alcohol dehydrogenase expression in both shoots and roots is conditioned by root growth environment.. Plant Physiol.

[83] Rankin PW, Jacobsen EL, Benjamin RC, Moss J, Jacobsen MK (1989). Quantitative studies of inhibitors of ADP-ribosylation in vitro and vivo.. J Biol Chem.

[84] Rohde A, Morreel K, Ralph J, Goeminne G, Hostyn V (2004). Molecular phenotyping of the pal1 and pal2 mutants of Arabidopsis thaliana reveals far-reaching consequences on phenylpropanoid, amino acid, and carbohydrate metabolism.. Plant Cell.

[85] Huang J, Gu M, Lai Z, Fan B, Shi K (2010). Functional analysis of the Arabidopsis PAL gene family in plant growth, development, and response to environmental stress.. Plant Physiol.

[86] Deikman J, Hammer PE (1995). Induction of Anthocyanin Accumulation by Cytokinins in Arabidopsis thaliana.. Plant Physiol.

[87] Devoto A, Ellis C, Magusin A, Chang HS, Chilcott C (2005). Expression profiling reveals COI1 to be a key regulator of genes involved in wound- and methyl jasmonate-induced secondary metabolism, defence, and hormone interactions.. Plant Mol Biol.

[88] Ogawa T, Ueda Y, Yoshimura K, Shigeoka S (2005). Comprehensive analysis of cytosolic Nudix hydrolases in Arabidopsis thaliana.. J Biol Chem.

[89] Bürkle A (2005). Poly(ADP-ribose). The most elaborate metabolite of NAD.. FEBS J.

[90] Baena-Gonzalez E, Rolland F, Thevelein JM, Sheen J (2007). A central integrator of transcription networks in plant stress and energy signaling.. Nature.

[91] Diaz Vivancos P, Wolff T, Markovic J, Pallardó FV, Foyer CH (2010). A nuclear glutathione cycle within the cell cycle.. Biochem J.

[92] Kornas A, Kuźniak E, Slesak I, Miszalski Z (2010). The key role of the redox status in regulation of metabolism in photosynthesizing organisms.. Acta Biochim Pol.

[93] Hughes NM, Reinhardt K, Field TS, Gerardi AR, Smith WK (2010). Association between winter anthocyanin production and drought stress in angiosperm evergreen species.. Journal of Experimental Botany.

[94] Munné-Bosch S, Alegre L (2003). Drought-induced changes in the redox state of alpha-tocopherol, ascorbate, and the diterpene carnosic acid in chloroplasts of Labiatae species differing in carnosic acid content.. Plant Physiol.

