# Supplementary figures and images for: Chemical PARP Inhibition Enhances Growth of Arabidopsis and Reduces Anthocyanin Accumulation and the Activation of Stress Protective Mechanisms
Source: PLoS One. 2012 May 25;7(5):e37287. doi: 10.1371/journal.pone.0037287 (PMC3360695; doi:10.1371/journal.pone.0037287)

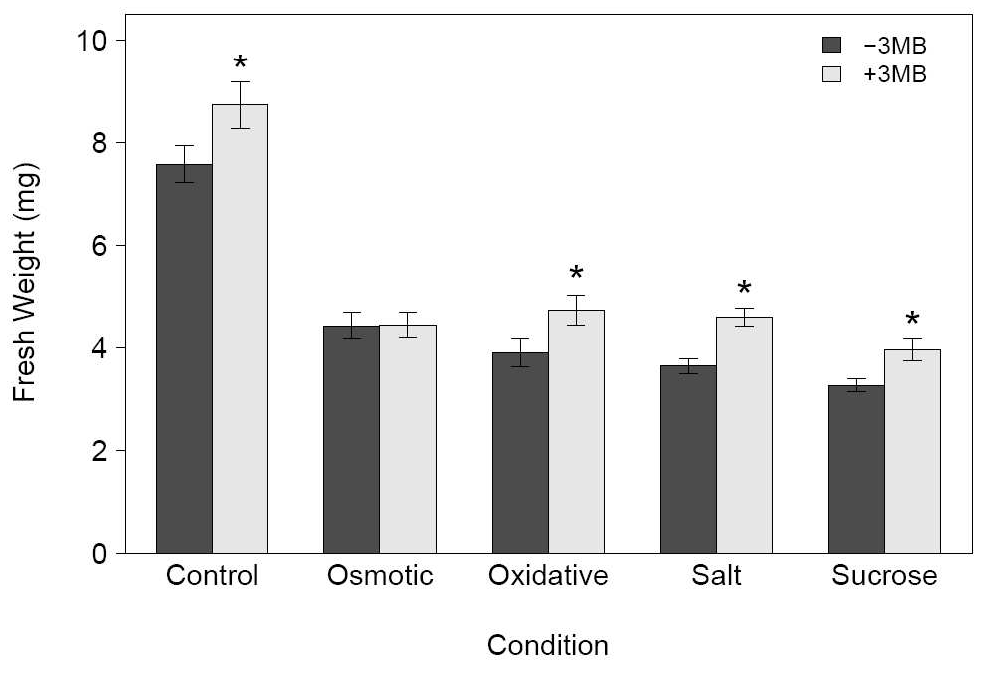

Supplement: Figure S1 — PARP inhibition leads to enhanced tolerance against long-term stress. Plants were grown for 14 days at 80–100 µE, 22°C on MS medium and subjected to five different conditions: control, oxidative stress (0.1 µM paraquat), sucrose stress (150 mM sucrose), osmotic stress (100 mM sorbitol) and salt stress (75 mM NaCl). The average fresh weight of individual plants was determined by weighing 32 pooled seedlings from each plate, with 5 replicates (plates) in each experiment repeated in three independent experiments (n = 15). Significant differences (P<0.05) between the seedlings grown with PARP inhibitor compared to those grown without in the same condition is indicated by an asterisk. (TIF) [file pone.0037287.s001.tif]

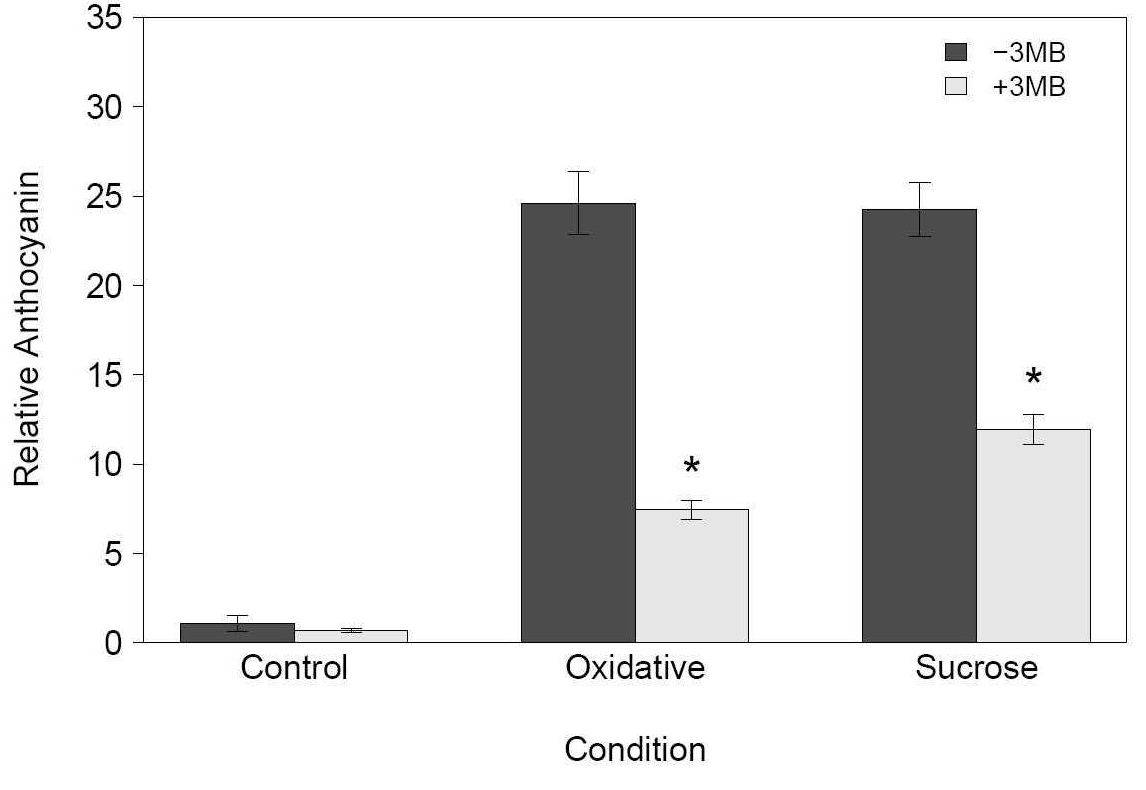

Supplement: Figure S2 — Chemically PARP inhibition reduces anthocyanin accumulation already at early stages. Arabidopsis seedlings (Col-0) were grown for 8 days at 80–100 µE, 22°C on MS medium and subjected to three conditions: control, oxidative stress (0.1 µM paraquat) or sucrose stress (150 mM sucrose) with (+3 MB) or without (−3 MB) the PARP inhibitor 3-Methoxy-benzamide in the media. The relative anthocyanin content is shown, data are combined from three independent experiments with 5 replicates in each experiment (n = 15). Asterisks indicate significant difference (P<0.05) in anthocyanin accumulation between seedlings grown with a PARP inhibitor compare to those without in the same condition. (TIF) [file pone.0037287.s002.tif]

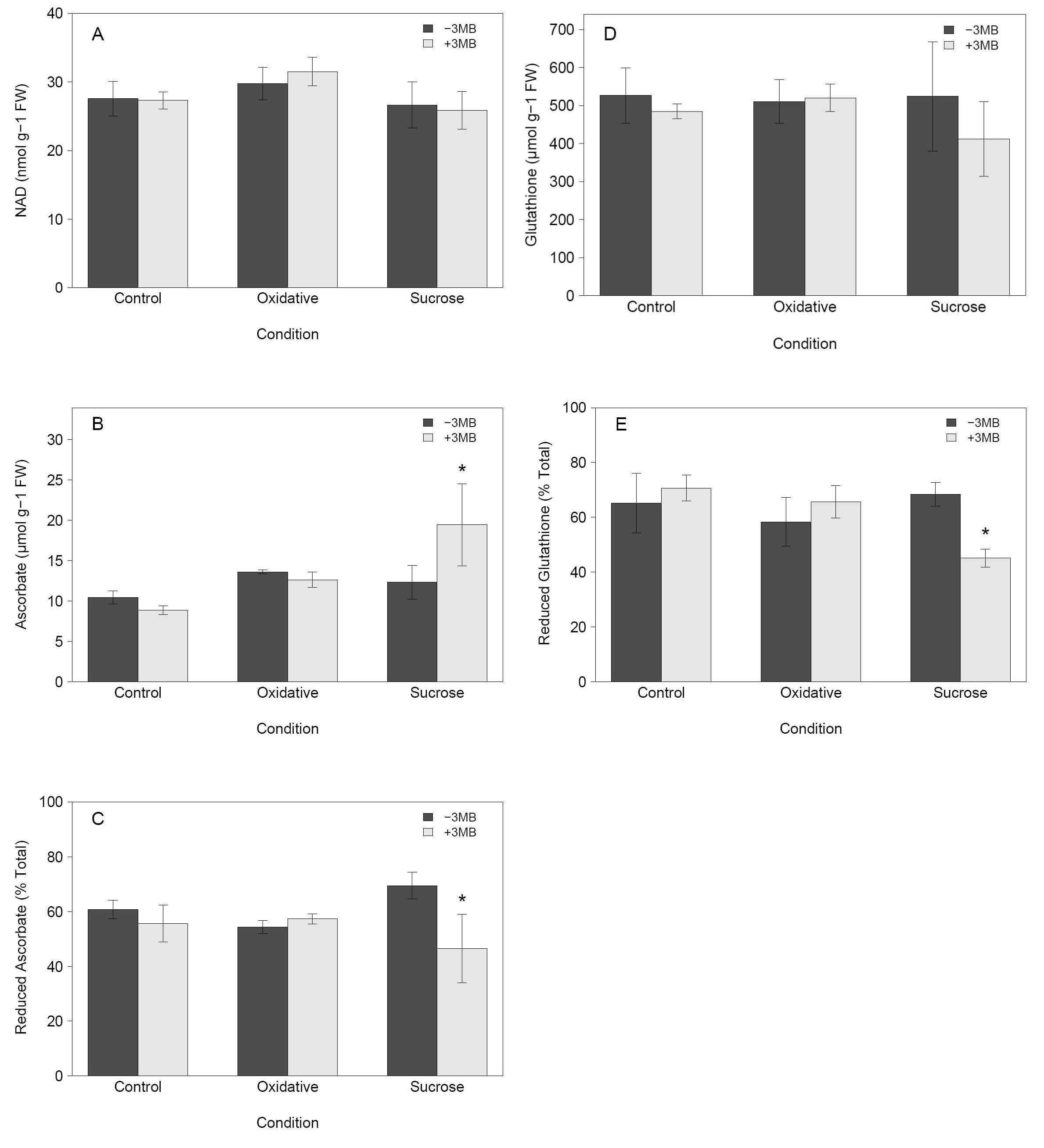

Supplement: Figure S3 — The effect of chemical PARP inhibition on cellular redox profiles at 8 days. Arabidopsis Col-0 seedlings were grown for 8 days at 80–100 µE, 22°C on MS media with (+3 MB) or without (−3 MB) the PARP inhibitor 3-Methoxy-benzamide (3 MB) and were subjected to three different treatments: control, oxidative stress (0.1 µM Paraquat) or sucrose stress (150 mM sucrose). Shown are (A) the NAD+ content, (B) the total ascorbate content, (C) the reduction level of the ascorbate, (D) the total glutathione and (E) the reduction of the total glutathione. Data are combined from three independent experiments with 1 or 2 replicates in each experiment (n = 4). Asterisks indicate significant difference (P<0.05) compared to Col-0 grown in the same condition without 3 MB. (TIF) [file pone.0037287.s003.tif]
